# Tumor-suppressive function of *UNC5D* in papillary thyroid cancer

**DOI:** 10.18632/oncotarget.21759

**Published:** 2017-10-10

**Authors:** Man-Man Zhang, Feng Sun, Bing Cui, Le-Le Zhang, Ya Fang, Yan Li, Rui-Jia Zhang, Xiao-Ping Ye, Yu-Ru Ma, Bing Han, Huai-Dong Song

**Affiliations:** ^1^ The Core Laboratory in Medicine Center of Clinical Research, Department of Endocrinology, Shanghai Ninth People's Hospital, Shanghai Jiaotong University School of Medicine, Shanghai 200011, China; ^2^ Department of Transfusion, The Hospital Affiliated to Jiangsu University, Zhenjiang 212001, China

**Keywords:** UNC5D, papillary thyroid carcinoma, tumor-suppressive function, lymph node metastasis, BRAF mutation

## Abstract

**Background:**

Studies have shown an association of the *UNC5D* gene with kidney and bladder cancer and neuroblastoma. We investigated whether *UNC5D* acts as a tumor suppressor in papillary thyroid carcinoma (PTC).

**Methods:**

Primary PTC tumors and matched normal thyroid tissues were obtained from 112 patients to detect *UNC5D* mRNA by real-time PCR. Genomic DNA sequencing was performed to detect *BRAF* mutation in PTC tumors. The association between *UNC5D* expression and clinicopathological data from PTC patients was reviewed retrospectively. PTC-derived cancer cell lines TPC-1 and K1 with stable transfection of *UNC5D* were used to investigate the functions of *UNC5D*. Flow cytometry, CCK-8, Transwell assay and scratch tests were used to examine cell cycle distribution, proliferation and migration.

**Results:**

The expression of *UNC5D* was significantly decreased in PTC compared with adjacent normal thyroid tissues. Lower *UNC5D* expression was significantly associated with aggressive tumor behaviors, such as lymph node metastasis and *BRAF* mutation. Overexpression of *UNC5D* significantly suppressed malignant cell behaviors, including cell proliferation and migration, as well as tumor growth *in vivo*.

**Conclusions:**

These findings suggest a potential tumor suppressor role of *UNC5D* in PTC progression; and provide insight into potential clinical relevance for the prognosis of PTC.

## INTRODUCTION

Thyroid carcinoma, the most frequent primary malignancy in the endocrine organs, has experienced a rapidly increasing incidence and prevalence in recent decades [1]. Originating from thyroid follicular cells, papillary thyroid carcinoma (PTC) accounts for the vast majority of thyroid cancers (80%) [2, 3]. PTC patients in early stages have a favorable prognosis, and the overall five-year survival rate is 95% [4]; however, when they occasionally de-differentiate into more aggressive and lethal thyroid cancers, the five-year survival rate drops to 59% in the late stage [3, 5]. Clinical variables including advanced stage, extra-thyroidal tumor extension, lymph node metastasis (LNM), and distant metastases have been associated with a poor prognosis in PTC [6]. However, the potential molecular mechanisms underlying the aggressive behavior of some papillary carcinomas that result in recurrence and metastatic lesions remain poorly understood [7].

Recent studies using next-generation sequencing (NGS) have indicated that thyroid carcinoma is predominantly driven by genetic and epigenetic alterations [8]. Previous studies showed that a major class of driver genes of PTC is involved in the mitogen activated protein kinase (MAPK) signaling pathway, including point mutations in *BRAF* and *Ras* [9–12], as well as fusions involving the *RET* [13] and *NTRK1* tyrosine kinases [14]. Mutations within the genes related in the phosphoinositide 3-kinase (PI3K) pathway, such as *PTEN, PIK3CA*, and *AKT1*, have also been reported at low frequencies [8]. Recently, increasing numbers of tumor suppressor genes (TSGs) have been identified in PTC, which implies that TSGs, such as *CHEK2*, *REC8*, *CCDC67*, and *PATZ1*, may play a vital role in tumorigenesis in PTC [3, 15–17]. Nevertheless, the molecular mechanisms of TGSs in PTC remain unknown, which makes further study necessary for understanding the pathogenesis of PTC.

Uncoordinated-5 (*Unc5*) receptors, including four homologues (*Unc5A-D*), were expressed extensively in multiple tissues and participated in an array of cell processes [18, 19]. Intriguingly, characterized as “dependence receptors”, *Unc5* receptors expression is strongly suppressed in most cancers [20, 21], presumably due to pro-apoptotic and antiangiogenic properties of *Unc5* signaling [22–24]. Overexpression of these receptors inhibits tumor cell anchorage-independent growth and invasion, which makes the *Unc5* receptors hypothesized to be putative tumor suppressors [25, 26].

*UNC5D*, a newly added member of the *Unc5* family [27], widely expressed in normal tissues, was frequently absent or attenuated in cancer cell lines and reported to be associated with multiple cancers including kidney cancer, neuroblastoma and bladder cancers [28–30]. However, to the best of our knowledge, whether *UNC5D* could act as a tumor suppressor gene in PTC remains unclear.

Considering this, we focused on the potential role of *UNC5D* in the development of PTC (see Supplementary Figure 1). First, *UNC5D* expression in papillary thyroid cancer versus adjacent noncancerous tissues was investigated, and the relationship between *UNC5D* and clinicopathological variates was explored. Subsequently, the implication of *UNC5D* downregulation in PTC was analyzed by monitoring altered PTC cell behaviors following restoration of its expression in otherwise silenced cells. Data thus acquired support a tumor-suppressive function of *UNC5D* in PTC. We put forth that our results will lead to improved clinical pathologic classification and management of PTC patients.

## RESULTS

### Loss or reduced expression of *UNC5D* in PTC and cell lines

**Figure 1:**
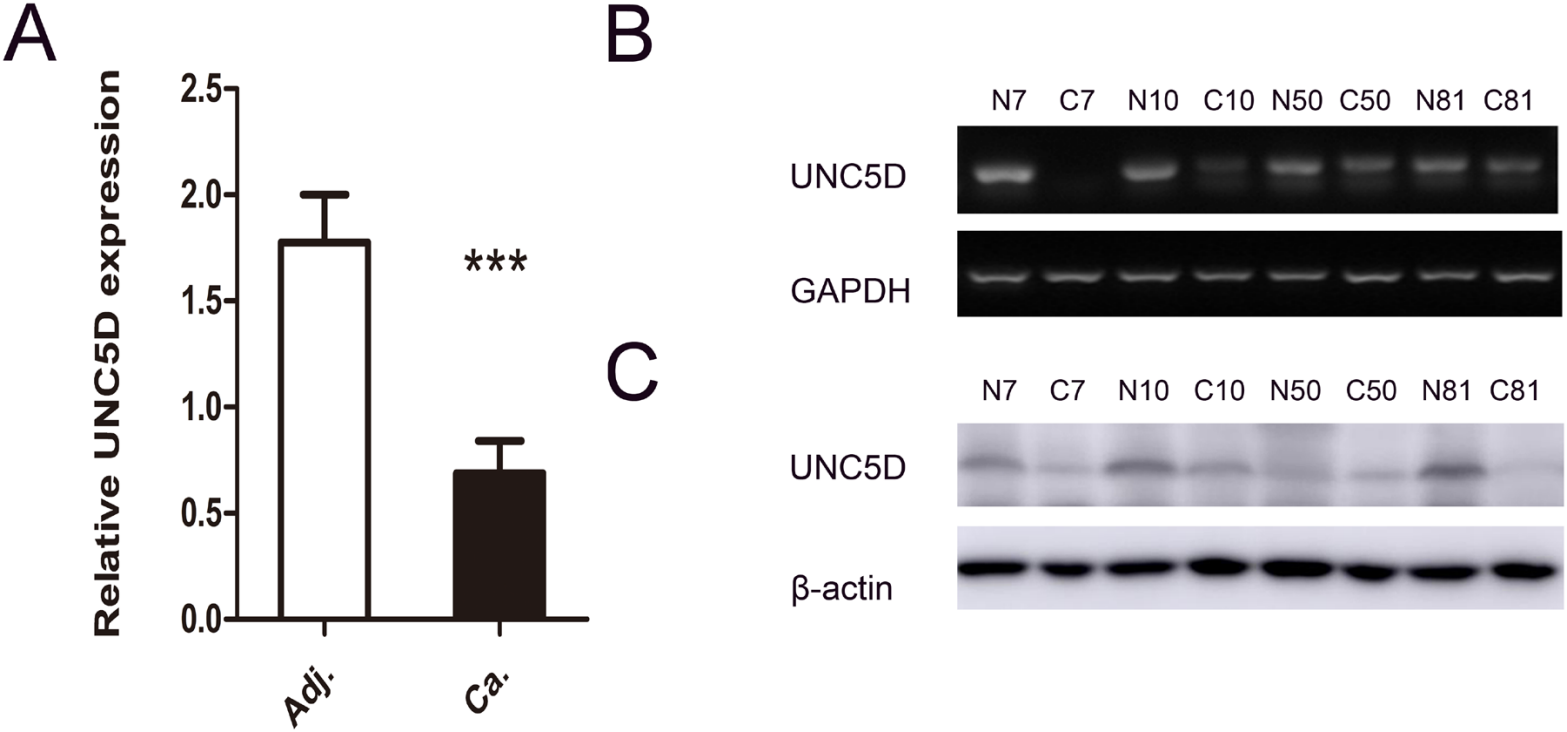
*UNC5D* is attenuated or silenced in PTC tissues **(A)** comparison of the relative expression levels of *UNC5D* in 112 paired PTC and adjacent noncancerous tissues as measured by real-time PCR. “^***^” indicates *P*< 0.01. **(B)** *UNC5D* mRNA expression in 4 representative pairs of PTC and adjacent noncancerous tissue as assessed by RT-PCR. **(C)** Western blot analysis of *UNC5D* protein in PTC and adjacent normal tissues. N, matched normal thyroid tissue; C, cancer tissue.

We performed quantitative real-time PCR and semi-quantitative reverse transcription PCR (RT-PCR) in 112 patients with primary PTC to explore the expression level of *UNC5D* mRNA in PTC. Quantitative real-time PCR analysis revealed that *UNC5D* mRNA expression was remarkably reduced in a large proportion of PTC tissues compared to adjacent noncancerous tissues. (Figure 1A). Such a pattern was subsequently verified using RT-PCR. As shown in Figure 1B, in a representative number of paired tissue specimens, the *UNC5D* mRNA expression level was weak in contrast to paired nonmalignant tissues. We further examined *UNC5D* protein expression in these tumors by Western blot. A similar pattern was seen with the expression of *UNC5D* protein (Figure 1C). Previous studies have indicated that hypermethylation of a CpG island in the promoter region together with loss of heterozygosity (LOH) may contribute to the *UNC5D* silencing in renal cancer [28]. These results suggested that deregulated expression of *UNC5D* might correlate with PTC tumorigenesis and development.

### Down-regulation of *UNC5D* expression is associated with *BRAF* (*V600E*) mutation and aggressive behaviors of papillary thyroid cancer

**Figure 2:**
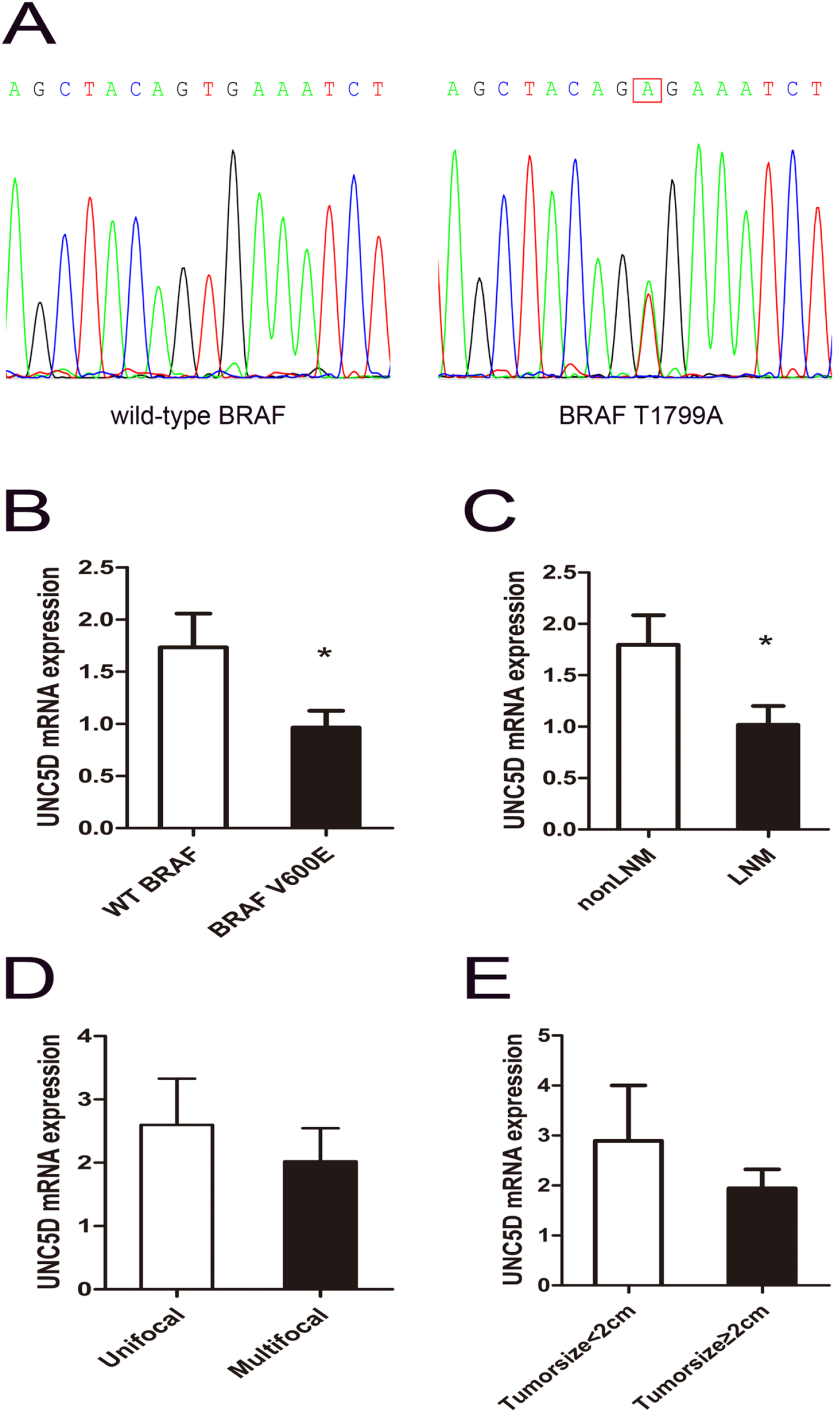
Aggressive tumor behaviors affected *UNC5D* mRNA expression levels in PTC **(A)** the representative electropherogram of the hotspot mutation in the *BRAF* gene. A case of PTC with the wild-type *BRAF* and a case with the *BRAF* T1799A mutation are shown. The red rectangle indicates the nucleotide where the mutation occurs. **(B-D)** contrast of *UNC5D* mRNA expression levels between subgroups divided by with or without the *BRAF*^V600E^ hotspot mutation (B), LNM or non-LNM(C), multifocality (D), and tumor size **(E)**. “^*^” indicates *P*< 0.05.

As a newly characterized tumor suppressor gene, *UNC5D* was recently reported to be mutated in non-small-cell lung cancer, uterine cancer and stomach cancer in a public dataset from The Cancer Genome Atlas (TCGA); however, the TCGA analysis of PTC implied that somatic mutations targeting *UNC5D* are uncommon in PTC, which was consistent with data from the Catalogue of Somatic Mutations in Cancer (COSMIC) database. *BRAF*^V600E^ was a commonly recognized hotspot mutation in PTC, and the *BRAF*^V600E^ mutant was sequenced by Sanger sequencing in our research. As shown in Table 1, *BRAF*^V600E^ was present in 69 of 110 patients. Figure 2A shows a representative electropherogram of the *BRAF* T1799A mutation. Compared to tumors without the *BRAF*^V600E^ hotspot mutation, the expression of *UNC5D* was significantly decreased in *BRAF* mutated tumors (Figure 2B). Interestingly, as illustrated in Figure 2C-2E, the *UNC5D* mRNA level in patients with LNM was lower than in the non-LNM group, while the expression level of *UNC5D* between subgroups divided according to multifocality or tumor size displayed no significant differences. Linear regression analysis of the association between clinicopathologic features and the expression of *UNC5D* revealed that in addition to BRAF mutation and LNM, no correlation between the reduction in *UNC5D* expression and the characteristics including age, gender, tumor size and multifocality existed (Table 2). As summarized in Table 3, logistic regression analysis showed that *UNC5D* expression was strongly associated with the occurrence of LNM.

**Table 1:** The clinical characteristics of study subjects

Characteristics	Number (%)
Number of patients	112
Tissue samples analyzed	224
Sex	
male	27
female	85
Age	
< 45y	49
≥ 45y	63
Tumor size^*^	
T1	63
T2	36
T3	8
Multifocality	34 (30%)
Lymph node metastasis	41 (38%)
Distant metastasis	0
Tumor mutation status	
BRAF V600E	69 (63%)

Tumor size were classified as tumor T status: T1 = ≤ 2 cm; T2 = > 2cm, but < 4 cm; T3 = ≥ 4 cm.

**Table 2:** Linear regression analysis of variables associated with the *UNC5D* expression in PTC subjects

(A)	Simple		Multiple	
	r	*P*	β	*P*
Sex(female/male)	0.078	0.415		
Age(years)	0.109	0.254		
Tumor size(cm)	0.011	0.909		
Multifocality	0.016	0.865		
*BRAF* V600E	0.267	0.005	0.229	0.014
LNM	0.202	0.036	0.233	0.013

**Table 3:** Logistic regression analysis of parameters associated with LNM

Parameters	Category	Univariate analysis	
		OR(95% CI)	*P*
Age (year)		1.005 (0.977-1.035)	0.726
Tumor size (cm)		1.052 (0.757-1.463)	0.763
*UNC5D*		1.371 (1.080-1.739)	0.009
Sex	Female/male	2.143 (0.772-5.947)	0.143
multifocality	Uni/multi-focal	1.310 (0.568-3.022)	0.527
*BRAF* status	WT/V600E	0.576 (0.251-1.321)	0.193

### Restoration decreased cell proliferation and colony formation

**Figure 3:**
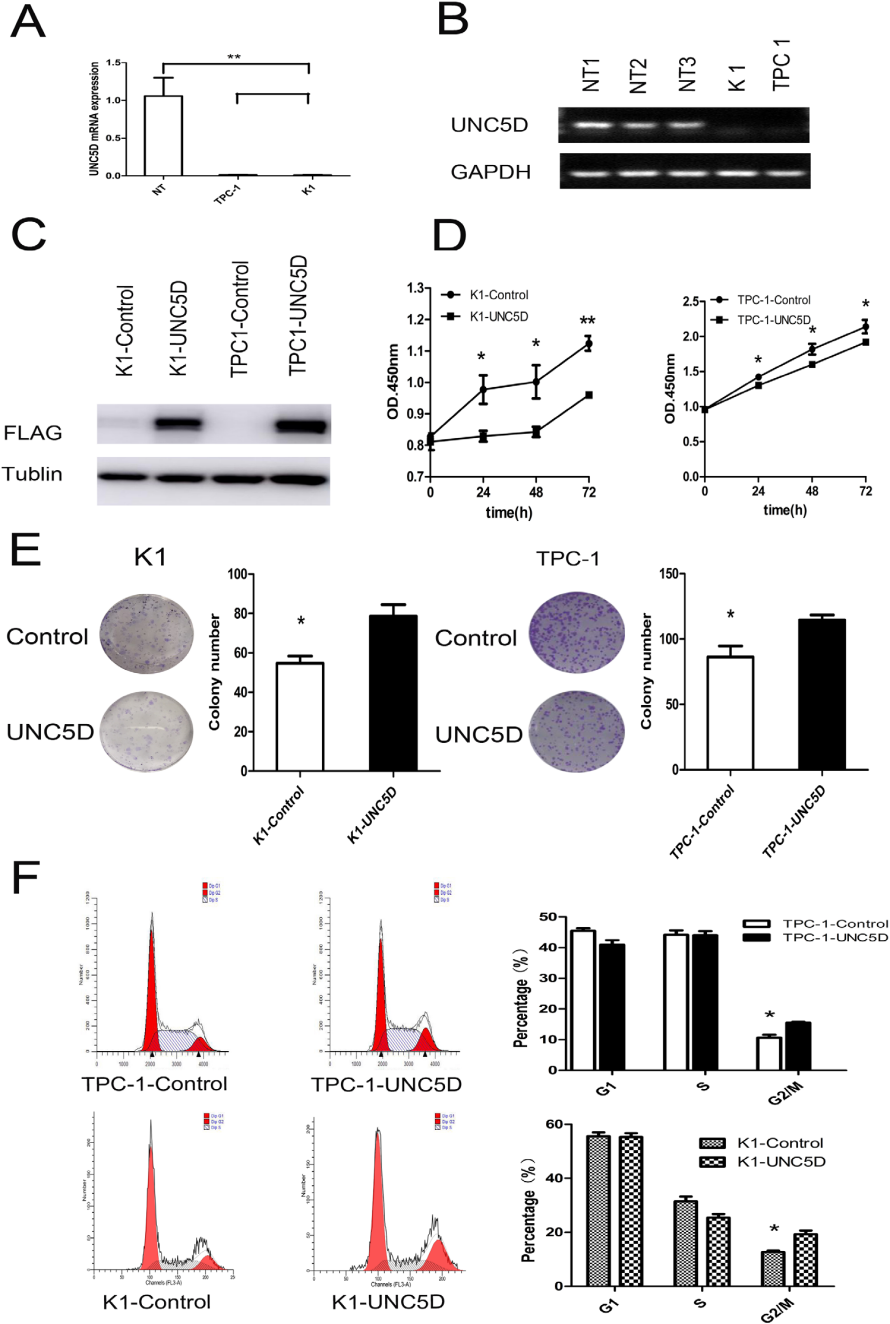
*UNC5D* inhibits PTC cell growth and cell cycle status K1 and TPC-1 cells were infected with control/*UNC5D*-expressing lentiviruses and monitored for growth using various assays. Each assay was repeated at least 3 times. **(A)** real time-PCR analysis of *UNC5D* in 2 PTC-derived cell lines (TPC-1 and K1) and 3 normal thyroid gland tissues (mean value of expression was set to 1). Means ± SEM of triplicate samples compared to each normal control are shown. NT = three normal thyroid tissues used as a control. **(B)** *UNC5D* mRNA expression in three normal thyroid tissues and 2 thyroid cancer cell lines as assessed by RT-PCR. **(C)** Western blot results show the protein expression of *FLAG* and *GAPDH*. **(D)** cell proliferation was analyzed using CCK-8. The results are presented as absorbance (OD) at 450 nm for triplicate wells. “^*^” indicates *P*< 0.05. **(E)** colony formation of K1 and TPC-1 cells in monolayer culture. Overexpression of *UNC5D* inhibited cell proliferation and the colony formation of K1 and TPC-1 cells in a monolayer culture, *P* <0.05. **(F)** the flow cytometric analysis of the cell cycle showed that *UNC5D* induced G2-M arrest in K1 and TPC-1 cells. The results quantified in the cell cycle analysis are shown as a percentage of the total number of cells. Data are expressed as the means±SEM of three independent experiments. “^*^” indicates *P*< 0.05.

To uncover whether the *UNC5D* acts as a tumor suppressor in PTC, the expression of *UNC5D* was further confirmed in two PTC-derived cancer cell lines (K1, TPC 1) by quantitative real-time PCR and RT-PCR. The results showed lower *UNC5D* expression levels compared to 3 normal thyroid gland tissues (Figure 3A-3B). To determine whether *UNC5D* could inhibit the proliferation or migration of thyroid cancer cells, we used a lentivirus to build the *UNC5D* overexpression and negative control cell lines. The effectiveness of the transfection was confirmed by Western blotting (Figure 3C). Cell proliferation and colony formation assays were then performed to observe the function of *UNC5D* in the proliferation of K1 and TPC-1 cells; the percentage of viable cells was detected at four time points (0, 24, 48, and 72 hours) (Figure 3D). It was found that the overexpressed *UNC5D* could inhibit cell proliferation in these two PTC-derived cancer cell lines. Cell colony formation was also significantly inhibited by *UNC5D* overexpression (Figure 3E). Cell-cycle analysis with *UNC5D* overexpressed K1 and TPC-1 cells both revealed an increase in G2-M phase (Figure 3F). Based on these results, we concluded that *UNC5D* might be a key mediator in the proliferation of K1 and TPC-1 cells.

### Overexpression of *UNC5D* inhibits PTC cells migration

**Figure 4:**
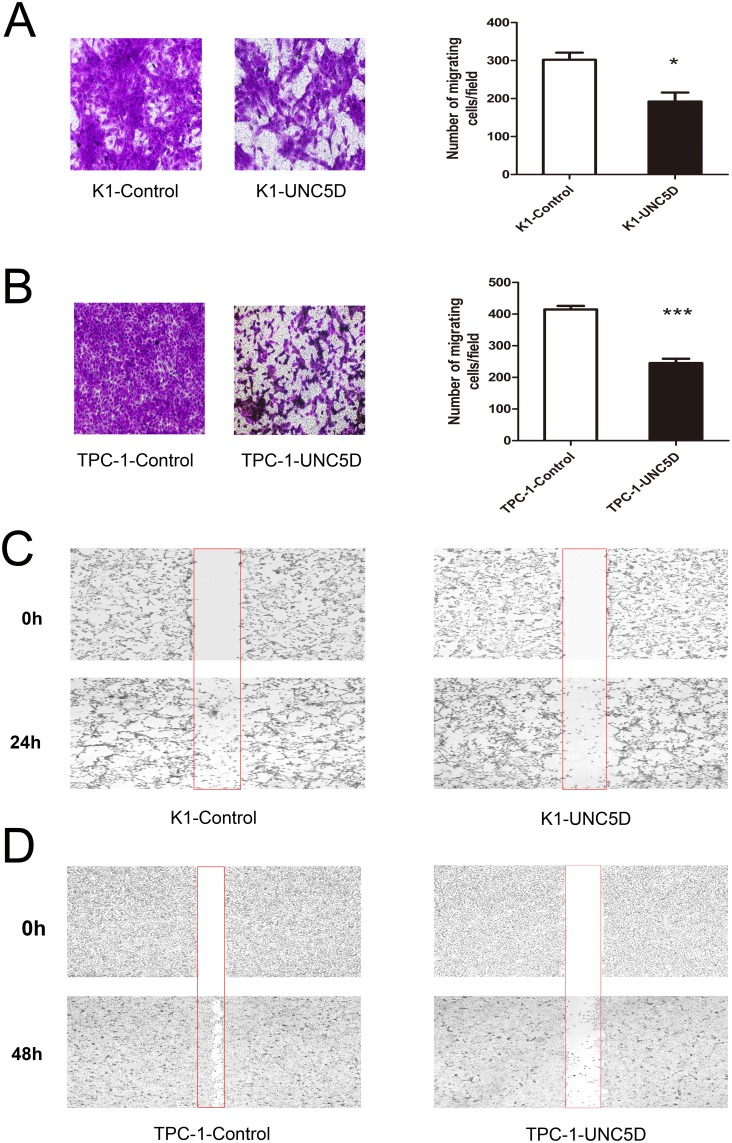
Effect of *UNC5D* on the migration of K1 and TPC-1 cells Transwell assay was performed to determine the migration ability of K1 **(A)** and TPC-1 cells **(B)**. Representative images showing cell migration in Transwell assay. The number of migration cells was counted in 6 randomly chosen fields and averaged for each of the triplicate wells. Data are expressed as the means±SEM of three independent experiments. “^*^” indicates *P* < 0.05, “^***^” indicates *P* < 0.01. Representative images showing the migration of K1 **(C)** and TPC-1 cells **(D)** in the scratch wound-healing assay at 2 points.

We next investigated whether *UNC5D* could inhibit the migratory abilities of K1 and TPC-1 cells by performing the Transwell assay and scratch wound healing assay. The results observed in all *UNC5D*-transfected clones, including K1 and TPC-1 cells, showed that overexpression of *UNC5D* led to a nearly 2-fold reduction in the number of cells crossing over the filter in the Transwell assay (Figure 4A-4B). By using a scratch wound-healing assay, we found that wound closure was retarded for *UNC5D*-overexpressing cells compared with the control cells transfected with only the empty vector (Figure 4C-4D).

### *UNC5D* inhibits tumor growth *in vivo*

**Figure 5:**
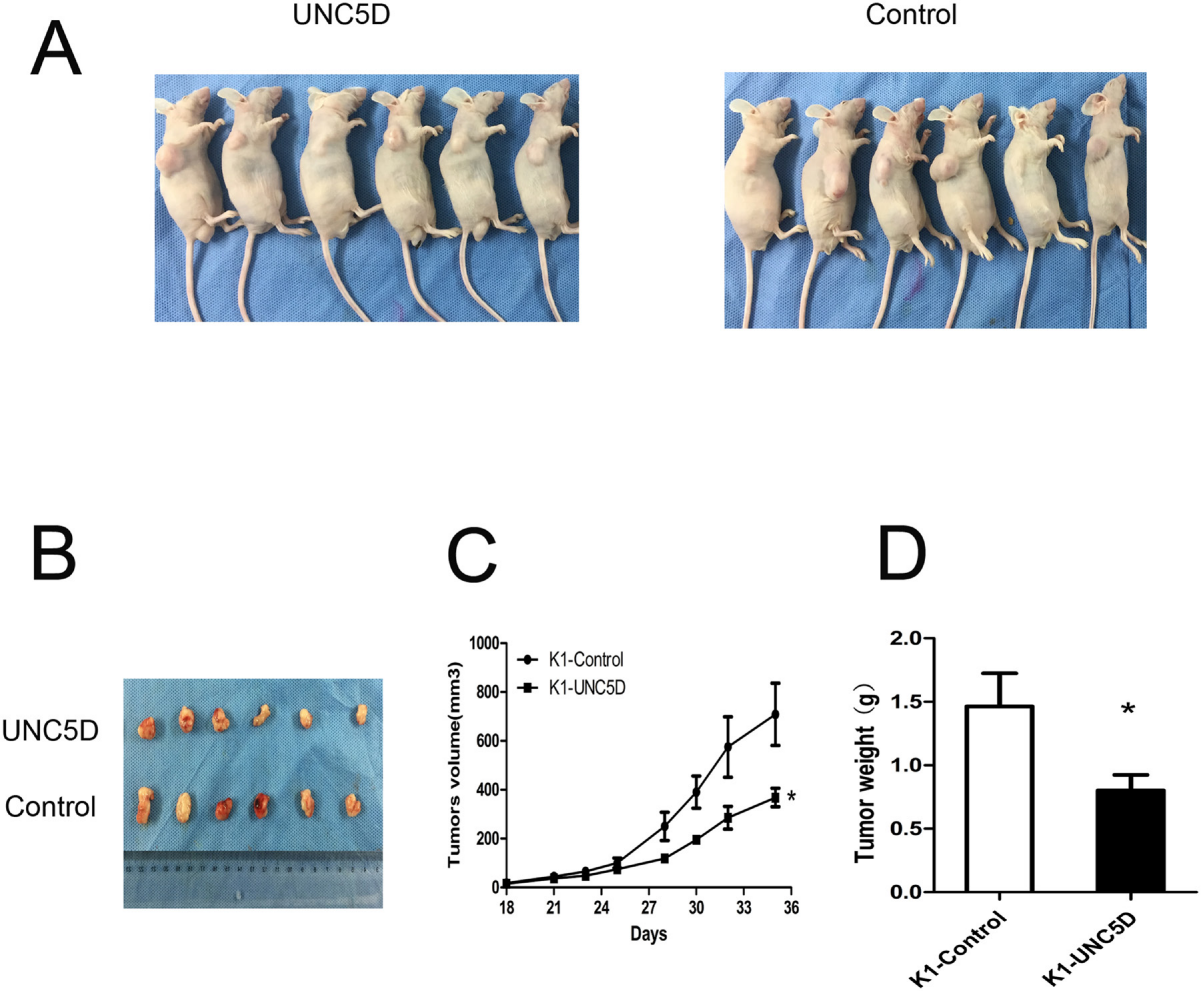
*UNC5D* inhibits PTC growth *in vivo* **(A)** twelve 4-5-week old male BALB/c nude mice were separated into 2 groups and injected with K1-Control cells or K1-*UNC5D* cells. **(B)** solid tumors were peeled from mouse subcutaneous tissue at 35 days post-injection. **(C)** tumor growth curves showed the volume of xenograft tumors changed in a time-dependent manner. **(D)** tumor weights were measured when mice were sacrificed. The means±SEM are reported. “^*^”, *P* < 0.05.

To further examine the effect of *UNC5D* on PTC cell growth *in vivo*, *UNC5D* overexpressed and control K1 cells were injected subcutaneously into 4-5-week-old male nude mice. Twelve mice were divided into 2 groups (Figure 5A), and tumors were peeled from the subcutis of nude mice (Figure 5B). Consistent with *in vitro* results, *UNC5D* significantly inhibited tumor growth *in vivo* through decreased tumor volume and weight in mice (*P*< 0.05) (Figure 5C-5D). These results confirmed *UNC5D* to be a novel tumor suppressor gene for PTC.

## DISCUSSION

*UNC5D* is the most recently identified member of the *UNC5* family. No information has been available concerning its biologic function in thyroid cancer to date. The current study, which focused on the role of *UNC5D*, showed for the first time that *UNC5D* expression was frequently reduced or lost in PTC tumors and implied its important tumor suppressor function in thyroid cancer cells.

Although the function of *UNC5D* is not yet completely clarified, previous studies have demonstrated that *UNC5D* expression was significantly higher in favorable neuroblastomas than in unfavorable ones, and higher *UNC5D* levels were correlated with longer survival time [29]. Lu et al. [28] reported that *UNC5D* was frequently absent or attenuated in cancer cell lines and primary renal cell carcinoma (RCC); ectopic *UNC5D* expression in a silenced renal cancer cell line dramatically inhibited the growth, migration and invasion of renal cancer cells. Meanwhile, genetic analysis showed that allelic imbalance significantly inhibits the *UNC5D* gene in unstable bladder tumors and that *UNC5D* may have important roles as a novel suppressor in bladder cancer via the *UNC5D/DAPK* pathway [30]. One study reported that *UNC5D* is induced during DNA damage-mediated apoptosis and is a direct transcriptional target of *p53* [31]. A genome-wide associated study has identified *UNC5D* as one of candidate genes associated with colon cancer predisposition [32]. Our previous study using multi-region sequencing also identified *UNC5D* as a significantly mutated gene in non-small-cell lung cancer and a late mutated event during tumorigenesis and progress [33]. However, the possible role of *UNC5D* in the tumorigenesis of PTC remains unstated.

PTCs are the most common tumors of the endocrine system, although the majority of them are effectively treated with surgery and radioactive iodine; however, of note, it presents with a high rate of LNM (45%), which results in a challenge in patients management [34, 35]. Therefore, it becomes urgent to expand our understanding of the pathogenic mechanisms of tumorigenesis and identify effective therapeutic targets for these refractory patients. Here, our study elicited that the expression level of *UNC5D* in PTC tumors was dramatically lower compared to normal adjacent tissues. Furthermore, a significant decrease in the *UNC5D* mRNA expression level was observed in PTC patients with a *BRAF*^V600E^ hotspot or LNM. Correlation analysis indicated a relatively good relationship between the expression level of *UNC5D* and the aggressive clinical factors, including *BRAF* mutation and LNM. The *BRAF^V600E^* mutation is common in PTC, and patients with PTC harboring the *BRAF^V600E^* mutation appear to display more aggressive clinical behavior [36]. LNM may be associated with poor prognosis; however, the mechanism of LNM remains unclear [37].

To assess the biological significance of *UNC5D* in thyroid cancer pathogenesis, we used a lentiviral-mediated *UNC5D* overexpression vector to effectively upregulate *UNC5D* expression in the PTC cell lines K1 and TPC-1. We found that overexpression of *UNC5D* significantly decreased the cellular capacity to proliferate, suggesting a tumor suppressor role of *UNC5D* in opposing the malignant transformation of PTC. Additionally, *UNC5D* overexpression also displayed a profound inhibitory effect on cell mobility, leading to reduced migratory activity in PTC cells. Cell cycle analysis in our study showed that overexpressed *UNC5D* could induce G2-M cell-cycle arrest in thyroid cancer cells, which was similar to the results that have been reported in primary renal cancer cells [28]. Cell cycle regulation is a common process crucial to the tumor formation [38]. In addition, one Netrin-1 receptor *DCC* induced G2-M arrest by inhibition of *Cdk1* [39]. Most recently, *UNC5B* was reported to inhibit proliferation and migration by inhibiting cell cycle progression at the G2-M phase in bladder cancer cells [40]. Further studies will test whether cell-cycle arrest is a common event initiated by *UNC5* receptors.

The finding of the present study of the association between the down-regulation of *UNC5D* and the *BRAFV600E* mutation in PTC was intriguing. It implicated that there existed a potential repellence between the activation of *BRAF* and *UNC5D* expression, and also suggested that the former might participate in the negative regulation of the latter, which indicated that *UNC5D* might be a new target gene of *BRAF^V600E^* in PTC. These findings might expand the genetic repertoire and provide a potential therapeutic target for thyroid cancer. Logistic regression analysis demonstrated a comparatively strong interrelation between *UNC5D* expression with the occurrence of LNM, which suggested that the potential role of *UNC5D* in the LNM of PTC and the specific mechanism requires clarification. Nevertheless, the clinicopathological data and the cellular functional data in the present study are sufficient to establish that *UNC5D* is a novel tumor suppressor gene in thyroid cancer and is associated with tumor aggressiveness.

In summary, this is the first study that highlighted the tumor suppressor potential of *UNC5D* in PTC, which may serve as a potential diagnostic and therapeutic target for PTC intervention. It also extends our current understanding of the mechanism of *UNC5D* in the pathogenesis of PTC.

## MATERIALS AND METHODS

### Subjects

A total of 112 patients with pathologically confirmed PTC were enrolled at the First Affiliated Hospital of Bengbu Medical College and Shanghai Ruijin Hospital between October 2013 and December 2014. PTC and adjacent normal thyroid tissue samples were obtained and immediately stored at -80°C. Histopathologic diagnoses were established according to the World Health Organization (WHO) classification and reviewed by two independent pathologists. This study was approved by the Research Ethics Committee of Shanghai Ninth People's Hospital. Demographic information for each patient is described in Supplementary Table 1.

### Detection of *BRAF* mutation in PTC

The *BRAF* T1799A mutation was analyzed using genomic DNA isolated from 112 cases of primary PTC tissue samples that were available for DNA isolation. Genomic DNA was extracted using a DNeasy Blood & Tissue Kit (QIAGEN, Valencia, CA, USA), according to the manufacturer's instructions. As DNA quality was suboptimal for subsequent PCR and sequencing in 2 samples, we excluded them and proceeded with the detection of *BRAF* gene mutation by direct genomic DNA sequencing analysis in 110 patients. The primers used for PCR were (5'-3'):TCTGCAGCATCTTCATTCCA (forward) and GCCTCAATTCTTACCATCCACA (reverse). After confirmation of the efficiency and quality of the amplification PCR by running the PCR products on a 2.0% agarose gel, the PCR products were purified and sequenced by BioSune Company (Shanghai, China).

### Cell culture

The human PTC cell lines TPC-1 and K1 were kind gifts from the Key Laboratory for Endocrine and Metabolic Diseases of the Chinese Health Ministry (Shanghai, China) [41]. TPC-1 cells were cultured in RPMI 1640 medium supplemented with 10% FBS (Gibco). K1 cells were cultured in DMEM (Gibco), MCDB (Sigma, Saint Louis, Missouri, USA), and F 12 (Gibco) (2:1:1) medium supplemented with 10% FBS (Gibco). These cell lines were maintained in a 5% CO2-humidified atmosphere at 37°C. All cell cultures were routinely passaged at 90-95% confluency.

### RNA extraction, reverse transcriptase PCR and real-time PCR

Total RNA was isolated from frozen stored tissue specimens and cells using Trizol reagent (Invitrogen) according to the manufacturer's instructions and then treated with DNase I at room temperature for 10 min to degrade possible contaminating genomic DNA. cDNA was made from 1 μg RNA templates using reverse transcriptase and oligo (dT) primer (Takara). Quantitative real-time PCR for a series of genes was performed in triplicate using the SYBR Green and ABI ViiA TM 7 Real-Time PCR System as previously described [42]. Data were analyzed and presented relative to the expression of the *GAPDH* housekeeping gene. The primer sequences used for real-time PCR are as follows: *UNC5D*: forward primer, 5'-GGGACACTGCCTCATTTCAT-3', reverse primer, 5'- CATGGAAGTCCTCCACCTGT-3'; GAPDH: forward primer, 5'- GAAGGTGAAGGTCGGAGTCA-3', and reverse primer, 5'- ATCTCGCTCCTGGAAGATGG-3'.

### Construction of lentiviral vectors and transduction

The full-length *UNC5D-3FLAG* cDNA clones were obtained from Generay (Shanghai, China). To construct TPC-1 and K1 cells stably expressing *3FLAG*-tagged *UNC5D*, a lentivirus-mediated infection system was used. For overexpression of *UNC5D*, DNA encoding *3FLAG*-tagged *UNC5D* was inserted into the multi-cloning site of the pLenti vector. The sequences of the primers were as follows: forward primer, 5'-CGGGATCCCGATGGGGAGAGCGGCGGC-3' and reverse primer, 5'-GCTCTAGAGCTTACTTGTCGTCATCGTCT-3'. Lentiviruses were subsequently produced by transiently co-transfecting HEK-293 cells with the pLenti-CMV-EGFP-PURO vector and the packaging vectors pLP1, pLP2, and plp/vsvg pMD2.G, using Lipofectamine 2000 (Invitrogen). At 48 h after transfection, media containing retroviruses were collected, filtered with 0.45 μm filters and used to infect cells in the presence of 8 μg/ml polybrene (Sigma). Infected cells were selected using 2 mg/ml puromycin (Sigma) and further maintained in growth medium. Overexpression of 3FLAG-tagged *UNC5D* was confirmed by real-time PCR and Western blot analysis.

### Western blotting

Cell pellets or thyroid tissue samples were lysed in sample buffer as previously described [43]. Total cellular protein concentrations were determined using a BCA assay kit (Beyotime Biotechnology, China). An equal amount of protein of approximately 30 μg was separated by sodium dodecyl sulfate-polyacrylamide gel electrophoresis (SDS-PAGE) and transferred onto a polyvinylidene fluoride (PVDF) membrane. They were then incubated with primary antibodies including anti-*GAPDH* (Cell Signal Technology), anti-*Flag* (Sigma-Aldrich), and anti-*UNC5D* (Santa Cruz) and subsequently with horseradish peroxidase-linked secondary antibodies. The signal was detected using enhanced chemiluminescence (Amersham Imager 600, USA).

### Cell proliferation

Cell proliferation was measured by using Cell Counting Kit-8 (CCK-8), according to the manufacturer's instructions. Each condition was repeated at least 3 times. Absorbance at 450 nm was measured on a microplate reader at the designated time points after treatment.

### Cell-cycle analysis

TPC-1 and K1 cells infected with lentiviruses encoding *UNC5D*, or *EGFP* alone, were harvested and fixed with ice-cold 70% ethanol. After washing with PBS, the cell pellet was resuspended in PBS containing 10 mg/mL propidium iodide (PI, Sigma Aldrich) and 500 mg/mL RNase A (Sigma-Aldrich) and incubated at 37°C for 30 minutes. Samples were then analyzed on a BD FACSCalibur.

### Colony formation assay

Five hundred infected TPC-1 and K1 cells were plated in a 6-well plate and cultured in medium for 10 days. Colonies were fixed with precooled methanol, and colonies were then stained with 0.5% (w/v) crystal violet for 30 minutes at room temperature, washed with PBS, photographed and counted.

### Wound-healing assay

Cell motility was determined by measuring the movement of cells close to an artificial wound. Cells were wounded with a 200 μL pipette tip, washed with PBS, and incubated in RPMI 1640 medium without FBS. The distances removed by cells were monitored by microscopy at the indicated time points.

### Transwell assay

For the migration assay, infected cells were seeded into the upper chamber of a Transwell with a fibronectin-coated filter (8-mm pore size, Corning Life Sciences). The bottom chamber contained medium containing 10% FBS. After a 20-hour incubation, cells adherent to the upper surface of the filter were removed using a cotton swab, and those attached to the bottom of the membranes were stained with crystal violet following fixation with methanol.

### Tumor xenografts model

Twelve BALB/c nude mice (4-5 weeks old, male) were randomly assigned to 2 groups. We injected infected K1-Control or K1-*UNC5D* cells (1 × 10^7^) subcutaneously into the right flank of BALB/c nude mice and measured the tumor dimensions by caliper every 2-3 days. The tumor volumes were calculated using the following formula: [length (mm) × width (mm) ×width (mm) × 0.5]. Upon termination, tumors were harvested and weighed. All experimental protocols conducted on the mice were performed in accordance with National Institutes of Health (NIH) guidelines and were approved by the Shanghai Jiaotong University Animal Care and Use Committee.

### Statistical analysis

All *in vitro* experiments were done in triplicate. Quantitative data are presented as individual data plots or as the means ± SEM of the 3 independent experiments with triplicate determinations. Statistically significant differences were determined by the 2-tailed unpaired Student's t-test. The correlations between gene expression and potential causative variables were evaluated with the Pearson or Spearman correlation. Logistic regression was used for the univariate analysis. These analyses were performed using SPSS 13.0 software (SPSS, Chicago, IL). *P* values <0.05 were considered statistically significant.

## SUPPLEMENTARY MATERIALS FIGURES AND TABLES





## Abbreviations

PTC: papillary thyroid cancer
TSG: tumor suppressor genes
*UNC5D*: unc-5 netrin receptor D
LNM: lymph node metastasis.
