# Supplementary material for: Tumor-suppressive function of UNC5D in papillary thyroid cancer
Source: Oncotarget. 2017 Oct 10;8(56):96126–38. doi: 10.18632/oncotarget.21759 (PMC5707086; doi:10.18632/oncotarget.21759)
Supplement: Supplementary file 1 [file oncotarget-08-96126-s001.pdf]

Tumor-suppressive function of *UNC5D* in papillary thyroid cancer

SUPPLEMENTARY MATERIALS

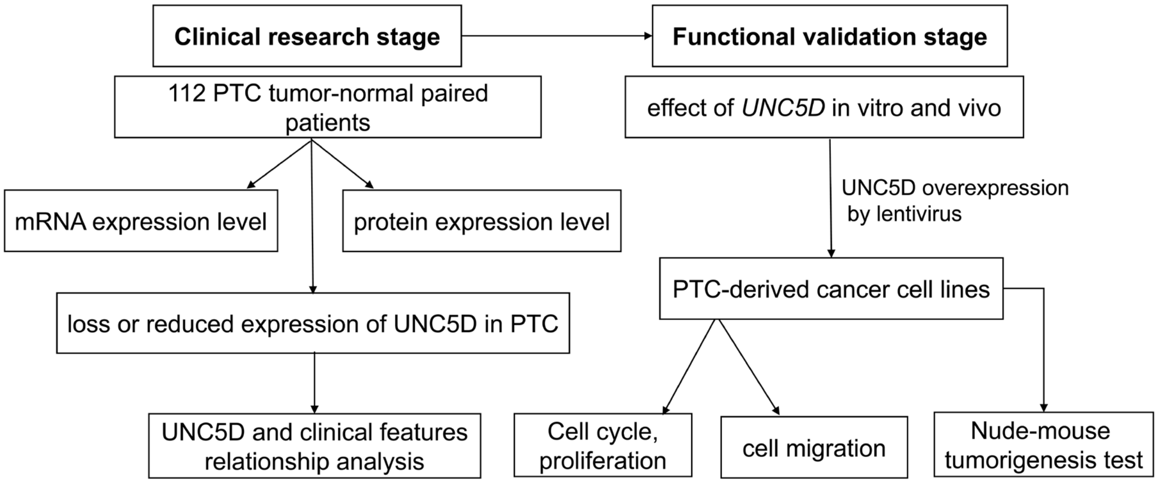

Supplementary Figure 1: A flowchart showing our study design.

Supplementary Table 1: Demographic information for 112 PTC patient

See Supplementary File 1
