# Supplementary material for: Tumor-suppressive function of UNC5D in papillary thyroid cancer
Source: Oncotarget. 2017 Oct 10;8(56):96126–38. doi: 10.18632/oncotarget.21759 (PMC5707086; doi:10.18632/oncotarget.21759)
Supplement: Supplementary file 2 [file oncotarget-08-96126-s002.docx]

Supplementary Table 1. Demographic information for 112 PTC patient

| Case NO. | Gender | Age （y) | Course（m） | Tumorsize(cm) | BRAF mutation | Multifocality | LNM | UNC5D(ΔCT) | |
| --- | --- | --- | --- | --- | --- | --- | --- | --- | --- |
|  |  |  |  |  |  |  |  | Cancer tissue | Normal tissue |
| 1 | Female | 38 | 3 | 2 | V600E | Multifocal | LNM | 10.58409 | 9.081509 |
| 2 | Male | 70 | 4 | 0.5 | Wildtype | Unifocal | NA | 9.993959 | 9.200847 |
| 3 | Female | 24 | 0.5 | 2 | V600E | Multifocal | NonLNM | 9.511413 | 9.789737 |
| 4 | Male | 40 | NA | 1.5 | V600E | Multifocal | NonLNM | 10.279333 | 8.212182 |
| 5 | Female | 27 | 0.23 | 1 | Wildtype | Multifocal | NonLNM | 8.464824 | 9.323259 |
| 6 | Male | 56 | 0.02 | 3 | Wildtype | Multifocal | NonLNM | 10.328216 | 11.507445 |
| 7 | Female | 52 | 3 | 0.8 | V600E | Unifocal | LNM | 16.526956 | 5.237761 |
| 8 | Female | 40 | 0.47 | 2 | V600E | Unifocal | NonLNM | 12.266994 | 10.279073 |
| 9 | Female | 45 | 2 | 2 | Wildtype | Multifocal | NonLNM | 6.951462 | 9.406074 |
| 10 | Male | 43 | 0.47 | 2.5 | V600E | Unifocal | LNM | 15.021466 | 8.769656 |
| 11 | Female | 24 | 24.33 | 0.3 | V600E | Unifocal | NonLNM | 11.21212 | 9.680314 |
| 12 | Female | 52 | 0.47 | 1.7 | V600E | Unifocal | LNM | 11.94066 | 10.825906 |
| 13 | Female | 23 | 0.1 | 1.5 | Wildtype | Multifocal | NonLNM | 9.393942 | 11.279307 |
| 14 | Female | 51 | 8 | 3.8 | Wildtype | Unifocal | NonLNM | 10.599772 | 8.429927 |
| 15 | Female | 41 | 6.8 | 1.6 | V600E | Unifocal | LNM | 10.566721 | 8.190195 |
| 16 | Male | 49 | 0.5 | 1.8 | V600E | Unifocal | LNM | 10.705796 | 11.137928 |
| 17 | Female | 28 | NA | 2.5 | V600E | Unifocal | NonLNM | 10.693822 | 9.749086 |
| 18 | Female | 35 | 0.3 | 3 | V600E | Unifocal | LNM | 12.012239 | 9.802592 |
| 19 | Female | 40 | 3 | NA | V600E | Unifocal | LNM | 12.868411 | 10.532691 |
| 20 | Male | 46 | 0.47 | 1.2 | NA | Unifocal | NA | 5.197827 | 9.819742 |
| 21 | Female | 47 | 0.67 | 0.4 | Wildtype | Unifocal | NonLNM | 10.793676 | 7.116579 |
| 22 | Male | 68 | NA | 3.5 | Wildtype | Unifocal | NonLNM | 10.02832 | 8.425995 |
| 23 | Female | 33 | 0.5 | 0.3 | Wildtype | Unifocal | NonLNM | 12.933549 | 7.765121 |
| 24 | Female | 41 | 4 | 0.4 | Wildtype | Multifocal | NonLNM | 8.608542 | 9.312346 |
| 25 | Female | 35 | NA | 3.5 | V600E | Unifocal | LNM | 13.903158 | 10.906267 |
| 26 | Female | 62 | 0.3 | 2.4 | Wildtype | Multifocal | LNM | 10.556576 | 7.620008 |
| 27 | Female | 60 | 3 | 2 | V600E | Unifocal | LNM | 10.145263 | 11.409738 |
| 28 | Female | 51 | 4 | 3 | V600E | Unifocal | NonLNM | 8.288593 | 9.653971 |
| 29 | Female | 55 | 1.7 | NA | V600E | Unifocal | LNM | 15.98674 | 7.697007 |
| 30 | Female | 44 | 0.23 | 2.5 | V600E | Unifocal | NonLNM | 11.442981 | 8.802412 |
| 31 | Male | 31 | NA | 1.6 | V600E | Multifocal | LNM | 12.06607 | 8.804515 |
| 32 | Female | 58 | NA | NA | Wildtype | Unifocal | NonLNM | 9.432909 | 10.363972 |
| 33 | Female | 65 | 3 | 3.6 | V600E | Unifocal | NonLNM | 12.078044 | 7.846758 |
| 34 | Female | 60 | 12.17 | 3.3 | V600E | Multifocal | LNM | 10.261477 | 7.16152 |
| 35 | Male | 23 | 1 | 2.3 | Wildtype | Multifocal | NonLNM | 7.768731 | 7.061881 |
| 36 | Female | 47 | 0.01 | 1.5 | Wildtype | Unifocal | NonLNM | 8.109842 | 9.344537 |
| 37 | Female | 31 | 0.23 | 3.3 | V600E | Unifocal | NonLNM | 11.906407 | 6.959904 |
| 38 | Female | 65 | 10 | 3.2 | V600E | Multifocal | LNM | 13.177356 | 9.716301 |
| 39 | Male | 58 | 0.1 | 0.2 | Wildtype | Unifocal | NonLNM | 9.067926 | 9.673354 |
| 40 | Female | 47 | 0.5 | 2 | V600E | Unifocal | LNM | 9.236032 | 8.633698 |
| 41 | Female | 55 | 6 | 2.4 | Wildtype | Unifocal | NonLNM | 10.074464 | 7.772948 |
| 42 | Male | 50 | 12.17 | 1.2 | Wildtype | Multifocal | LNM | 10.210783 | 8.558635 |
| 43 | Female | 31 | 0.3 | 2 | V600E | Unifocal | NonLNM | 11.009394 | 6.835971 |
| 44 | Female | 34 | 0.17 | 5.3 | Wildtype | Unifocal | NonLNM | 10.27145 | 10.704825 |
| 45 | Female | 65 | 5 | NA | V600E | Multifocal | NonLNM | 14.103773 | 9.031247 |
| 46 | Male | 52 | 0.43 | 4.6 | V600E | Multifocal | NonLNM | 10.863873 | 9.264426 |
| 47 | Male | 30 | 2.75 | 4.3 | V600E | Multifocal | NonLNM | 10.120912 | 11.546197 |
| 48 | Female | 40 | 4 | 3.8 | V600E | Unifocal | NonLNM | 10.710097 | 7.841952 |
| 49 | Female | 49 | 0.33 | 5 | V600E | Multifocal | LNM | 11.837626 | 9.474022 |
| 50 | Female | 42 | 3 | 0.7 | Wildtype | Unifocal | NonLNM | 12.231748 | 5.73652 |
| 51 | Female | 68 | 70 | 2.7 | V600E | Multifocal | NonLNM | 12.890082 | 9.645529 |
| 52 | Male | 66 | 1 | 2.1 | NA | Unifocal | NA | 6.83484 | 11.108491 |
| 53 | Male | 40 | 0.23 | 2 | V600E | Unifocal | NonLNM | 9.15281 | 9.536274 |
| 54 | Female | 29 | 0.5 | 4 | V600E | Multifocal | LNM | 11.639082 | 7.001876 |
| 55 | Female | 67 | 12.5 | 3.5 | Wildtype | Unifocal | LNM | 8.065057 | 7.459835 |
| 56 | Female | 78 | 0.06 | 1.5 | V600E | Unifocal | LNM | 9.401634 | 9.000087 |
| 57 | Female | 35 | 6 | 2 | V600E | Unifocal | NonLNM | 9.357177 | 8.952493 |
| 58 | Female | 54 | 0.23 | 1.5 | Wildtype | Multifocal | LNM | 12.194017 | 5.565063 |
| 59 | Female | 70 | 1 | 2.5 | V600E | Multifocal | NonLNM | 9.694071 | 8.075603 |
| 60 | Female | 30 | 2 | 0.8 | V600E | Unifocal | LNM | 10.65309 | 10.13225 |
| 61 | Female | 49 | NA | NA | V600E | Unifocal | LNM | 12.795712 | 8.541338 |
| 62 | Female | 55 | 3 | 1.2 | Wildtype | Unifocal | NonLNM | 11.380969 | 11.244145 |
| 63 | Male | 50 | 0.33 | 2 | Wildtype | Unifocal | NonLNM | 9.978575 | 8.545548 |
| 64 | Female | 31 | 0.17 | 0.5 | V600E | Unifocal | NonLNM | 7.187348 | 8.549384 |
| 65 | Female | 59 | 2 | 0.7 | V600E | Multifocal | NA | 16.433345 | 17.219271 |
| 66 | Male | 30 | 1 | 2 | Wildtype | Unifocal | NonLNM | 12.805601 | 9.232841 |
| 67 | Male | 57 | 0.7 | 0.6 | Wildtype | Unifocal | LNM | 11.329641 | 10.481586 |
| 68 | Female | 22 | 0.47 | 1.2 | Wildtype | Unifocal | LNM | 8.713344 | 10.409061 |
| 69 | Female | 52 | 0.1 | 0.3 | V600E | Unifocal | NonLNM | 11.681675 | 12.265424 |
| 70 | Female | 44 | 0.07 | 3.2 | V600E | Unifocal | NonLNM | 8.651949 | 10.018844 |
| 71 | Female | 33 | 6 | 1.5 | Wildtype | Unifocal | NonLNM | 11.581155 | 9.677518 |
| 72 | Female | 58 | 1 | 2 | V600E | Unifocal | NonLNM | 10.535566 | 7.843858 |
| 73 | Female | 36 | 1.5 | 1.5 | V600E | Unifocal | NonLNM | 13.009442 | 8.805248 |
| 74 | Female | 53 | 1 | 1.6 | Wildtype | Unifocal | NonLNM | 8.529748 | 9.558385 |
| 75 | Female | 57 | 5 | 1.7 | V600E | Unifocal | LNM | 14.06846 | 10.482011 |
| 76 | Male | 39 | NA | 1.3 | V600E | Unifocal | NonLNM | 10.8647 | 8.107956 |
| 77 | Female | 34 | 0.13 | 1.5 | V600E | Unifocal | LNM | 9.739573 | 9.27411 |
| 78 | Female | 47 | NA | 0.8 | V600E | Unifocal | NonLNM | 11.351933 | 10.600024 |
| 79 | Female | 32 | 0.1 | 0.2 | Wildtype | Multifocal | LNM | 10.2325 | 10.763979 |
| 80 | Female | 26 | 36.5 | 2 | V600E | Unifocal | NonLNM | 9.274091 | 10.111214 |
| 81 | Female | 31 | 1 | 4.2 | Wildtype | Unifocal | LNM | 12.695397 | 6.871567 |
| 82 | Female | 64 | 2 | 1 | Wildtype | Multifocal | NonLNM | 9.302692 | 7.171537 |
| 83 | Female | 49 | 0.5 | 4.3 | V600E | Unifocal | NonLNM | 10.698578 | 9.236753 |
| 84 | Female | 57 | 2 | 4 | Wildtype | Unifocal | NonLNM | 12.361703 | 8.297342 |
| 85 | Female | 60 | 12.17 | 3 | V600E | Multifocal | LNM | 12.89195 | 9.345761 |
| 86 | Female | 59 | 0.67 | 2 | V600E | Unifocal | NonLNM | 11.374175 | 5.608738 |
| 87 | Male | 51 | 0.23 | 2.2 | V600E | Multifocal | NonLNM | 12.329897 | 8.343325 |
| 88 | Male | 42 | 5 | 3.5 | V600E | Unifocal | LNM | 9.461418 | 6.841952 |
| 89 | Female | 35 | 1 | 1.3 | V600E | Multifocal | LNM | 11.758743 | 10.139249 |
| 90 | Female | 28 | 0.93 | 1.2 | V600E | Unifocal | LNM | 12.53284 | 10.956087 |
| 91 | Female | 47 | 1 | 3.1 | V600E | Unifocal | NonLNM | 8.779592 | 8.916617 |
| 92 | Male | 45 | 0.23 | 1.5 | V600E | Unifocal | NonLNM | 11.818427 | 11.441678 |
| 93 | Female | 45 | 11.87 | 2.2 | V600E | Unifocal | LNM | 9.175666 | 9.031247 |
| 94 | Female | 46 | NA | 2 | Wildtype | Unifocal | LNM | 11.226027 | 9.605559 |
| 95 | Female | 50 | 1 | 1 | Wildtype | Unifocal | NonLNM | 12.250686 | 8.631737 |
| 96 | Female | 48 | 0.45 | 2.5 | Wildtype | Unifocal | LNM | 10.455794 | 9.682824 |
| 97 | Female | 27 | 6 | 4.2 | Wildtype | Multifocal | LNM | 10.403313 | 11.316151 |
| 98 | Male | 62 | 1 | 2.6 | Wildtype | Multifocal | NonLNM | 14.004427 | 8.27075 |
| 99 | Male | 40 | 0.23 | 0.3 | V600E | Multifocal | NonLNM | 13.259266 | 7.787283 |
| 100 | Male | 46 | 24.33 | 2.8 | Wildtype | Unifocal | NonLNM | 10.47656 | 10.561392 |
| 101 | Female | 33 | 0.03 | 2.5 | V600E | Unifocal | NonLNM | 11.941984 | 7.090123 |
| 102 | Female | 39 | 0.23 | 2.8 | V600E | Multifocal | LNM | 11.368244 | 6.900267 |
| 103 | Male | 17 | 2 | 2 | V600E | Unifocal | NonLNM | 11.847909 | 8.429927 |
| 104 | Female | 40 | 8 | 1 | Wildtype | Multifocal | NonLNM | 11.52368 | 10.690279 |
| 105 | Male | 59 | 17 | 6 | V600E | Unifocal | NonLNM | 11.17352 | 10.890269 |
| 106 | Female | 38 | 4.3 | 2 | V600E | Unifocal | NonLNM | 11.381522 | 9.908428 |
| 107 | Female | 76 | 0.33 | 2.1 | V600E | Unifocal | LNM | 17.151423 | 10.47335 |
| 108 | Female | 75 | 6 | 3.4 | V600E | Unifocal | NonLNM | 10.44961 | 9.30109 |
| 109 | Female | 53 | NA | 1 | Wildtype | Unifocal | LNM | 11.151034 | 13.118692 |
| 110 | Female | 55 | 6 | 2 | Wildtype | Unifocal | NonLNM | 9.68858 | 9.15851 |
| 111 | Female | 46 | 0.13 | 0.5 | V600E | Unifocal | NonLNM | 10.965778 | 8.248633 |
| 112 | Female | 74 | 6 | 2 | V600E | Multifocal | NonLNM | 10.116185 | 6.803438 |

The *UNC5D* mRNA expression level of PTC tumor and normal tissues were exhibited as ΔCT, normalized to GAPDH;

y, years; LNM, lymph node metastasis; NA, not available.
